# Novel Strategies for Yuba Quality Improvement: Protein Modification Based on Physical Fields

**DOI:** 10.3390/foods14061033

**Published:** 2025-03-18

**Authors:** Wenchao Liu, You Tian, Lijuan Wang, Rui Hu, Yan Zhang, Linlin Li, Weiwei Cao, Xu Duan, Guangyue Ren

**Affiliations:** 1 College of Food and Bioengineering, Henan University of Science and Technology, Luoyang 471000, China; wen_chaoliu@163.com (W.L.); spxyyjs_xwgl@163.com (Y.Z.); linlinli2020@126.com (L.L.); caoweiwei@haust.edu.cn (W.C.); duanxu_dx@163.com (X.D.); 2 Henan Agricultural Products Processing Equipment Engineering Research Center, Luoyang 471000, China; 3 Yunnan Key Laboratory of Plateau Food Advanced Manufacturing, Kunming 650500, China; 4 School of Food Science and Engineering, South China University of Technology, Guangzhou 510641, China; hurui2023@163.com; 5 Academy of Contemporary Food Engineering, South China University of Technology, Guangzhou Higher Education Mega Center, Guangzhou 510006, China; 6 College of Basic Medical Science, Ningxia Medical University, Yinchuan 750004, China; mnn717@163.com

**Keywords:** yuba, physical field protein modification, quality improvement

## Abstract

This study investigated the effects of physical field protein modification methods on the mechanical properties, color, rehydration performance, thermal stability, and sensory quality of yuba. The results showed that all three modification methods shortened the drying time of yuba, and each method enhanced the tensile strength and thermal stability of yuba. Yuba treated with microwave–vacuum for 10 min demonstrated the best performance in terms of tensile strength, elongation, color, and overall sensory score, making it the optimal method for the physical field modification of yuba. In addition, microwave–vacuum treatment led to better rehydration performance, thermal stability, and a faster rehydration rate. Through the analysis of the microstructure of yuba as well as its protein secondary and tertiary structures, it was found that microwave–vacuum treatment can maintain the tissue network structure of yuba while promoting more heat-induced protein conformational changes, showing a greater increase in the content of *β*-sheets, which contribute to enhancing the tensile strength and water-holding capacity of yuba, thereby improving its product quality.

## 1. Introduction

Yuba, a millennium-old traditional Chinese soybean delicacy, is produced through an intricate process of soybean soaking, grinding, filtration, boiling, interfacial polymerization, and drying [1]. As a plant-based protein superfood, yuba contains all nine essential amino acids in nutritionally balanced proportions, complemented by beneficial lipids, micronutrients, and vitamins [2]. This unique composition positions yuba as a sustainable alternative to animal-derived proteins in modern dietary systems. The exceptional nutritional profile and textural versatility of yuba stem from its hierarchical protein architecture, where thermally induced protein denaturation and subsequent aggregation drive the formation of anisotropic fibrous networks during its manufacturing [3]. Crucially, the mesoscopic organization of these protein assemblies governs the material properties of yuba, including tensile strength, rehydration capacity, and thermal resilience [4].

Current strategies for yuba quality enhancement predominantly focus on modulating soybean protein conformation through chemical or enzymatic approaches. While covalent modification techniques such as glycosylation and phosphorylation can improve protein functionality [5], they often compromise nutritional integrity through reagent residues or uncontrolled proteolysis [6]. Enzymatic treatments, though more specific, face scalability challenges due to cost constraints and batch-to-batch variability [7]. In contrast, physical field-based protein modification emerges as a green chemistry paradigm, leveraging thermal/non-thermal energy inputs (e.g., ultrasonic cavitation, dielectric heating, and vacuum-assisted microwave irradiation) to induce controlled structural transitions without chemical additives [8]. Recent advances in food physics have revealed that precisely tuned physical fields can reconfigure protein quaternary structures through mechanisms like dipole realignment, interfacial tension modulation, and controlled free radical generation [9]. However, the translation of these principles to yuba processing remains underexplored, particularly regarding the spatiotemporal interplay between energy field parameters and protein network evolution.

This study pioneers a multimodal physical field approach for yuba quality optimization, systematically investigating the impact of ultrasound, microwave, and microwave–vacuum synergies on wet yuba matrices. Using a combination of molecular spectroscopy (FTIR and XRD), micro-rheology, and fractal dimension analysis, we elucidate how energy field-induced protein restructuring governs macroscopic product characteristics. The proposed strategy demonstrates exceptional industrial compatibility, requiring no chemical inputs. These findings advance the fundamental understanding of thermal/non-thermal protein engineering and provide a scalable platform for manufacturing premium plant-based protein foods.

## 2. Materials and Methods

### 2.1. Materials and Reagents

The soybeans were purchased from Luoyang Dazhang Supermarket. Disodium hydrogen phosphate (analytical grade), potassium dihydrogen phosphate (analytical grade), and potassium chloride (analytical grade) were purchased from Tianjin Deen Chemical Reagent Co., Ltd. (Tianjin, China). Barium chloride (analytical grade) was purchased from Jiangsu Qiangsheng Functional Chemicals Co., Ltd. (Changshu, China).

### 2.2. Experimental Methods

#### 2.2.1. Preparation of Wet Yuba

Building upon systematic optimization of preliminary trials, a standardized yuba fabrication protocol was established through the following critical control parameters: Fresh non-GMO soybeans (Glycine max L., 2023 harvest, Luoyang Dazhang Supermarket, Luoyang, China) were precisely metered (100.0 ± 0.5 g) using an analytical balance (ME204E, Mettler Toledo, Greifensee, Switzerland), followed by triple-rinse purification with reverse osmosis water; hydration treatment commenced through 12 h immersion in deionized water (25 ± 1 °C, 1:5 *w/v* ratio) under nitrogen atmosphere to prevent oxidative degradation; lastly, the hydrated beans were then wet-milled using a high-shear blender (Ultra-Turrax UTL-2000, IKA Werke GmbH & Co. KG, Staufen, Germany) at 15,000 rpm for 3 × 2 min cycles with ice bath intervals to minimize thermal denaturation.

The resultant slurry underwent sequential processing:

Liquid–solid separation: Dual-stage filtration through a 100 μm nylon mesh followed by vacuum-assisted Büchner filtration (0.45 μm cellulose membrane)

Thermal stabilization: Isobaric boiling (100 °C, 101.3 kPa) for precisely 120 s in a jacketed stainless steel reactor (KGW-3000, Kori, Germany) with magnetic stirring (300 rpm)

Interfacial polymerization: Controlled film formation in a thermostatically regulated water bath (85.0 ± 0.5 °C, Julabo CF41, Germany) using a custom-designed skimming apparatus with automated temporal control (±1 s resolution)

Critical quality attributes were quantified exclusively on secondary skimming-phase products (15 min interval specimens) to ensure inter-batch consistency.

#### 2.2.2. Protein Modification Strategies

A total of ten protein modification strategies were developed in this study, as shown in Table 1.

#### 2.2.3. Determination of Moisture Content on a Wet Basis

The moisture content of the sample was determined through drying in a 105 °C hot air drying oven (Model 101, Beijing Kewei Yongxing Instrument Co., Ltd., Beijing, China) until a constant weight was reached, with the weight difference between the pre-drying and post-drying samples not exceeding 2 mg. This procedure was repeated three times, and the average value was used for calculation. The moisture content on a wet basis was then computed using Equation (1).

(1) $$\omega_{t}=\frac{m_{t}-m_{g}}{m_{t}}\times 100$$

where *ω_t_* is the moisture content on a wet basis, %; *m_t_* is the mass of the sample at time *t*, g; and *m_g_* is the dry weight of the sample, g.

#### 2.2.4. Determination of Moisture Content on a Dry Basis and the Drying Rate

The moisture content on a dry basis and the drying rate were calculated according to Equations (2) and (3).

(2) $$X_{t}=\frac{m_{t}-m_{g}}{m_{g}}$$

where *X_t_* is the moisture content on a dry basis, g∙g^−1^; *m_t_* is the mass of the sample at time *t*, g; and *m_g_* is the dry weight of the sample, g.

(3) $$D_{R}=\frac{X_{t_{1}}-X_{t_{2}}}{t_{2}-t_{1}}$$

where *D_R_* is the drying rate, g∙g⁻^1^∙h⁻^1^; *X_t_*_1_ and *X_t_*_2_ are the moisture contents on a dry basis at times *t*_1_ and *t*_2_, g∙g^−1^; and *t*_1_ and *t*_2_ are the drying times, h.

#### 2.2.5. Analysis of the Mechanical Properties of Yuba

The yuba was equilibrated in a saturated barium chloride solution for 12 h, followed by cutting into 2 cm × 6 cm strips. The thickness and width were measured at five randomly selected points using a vernier caliper, and the average values were calculated. The total length of the yuba before and after fracture was also recorded. The mechanical properties of the samples were measured using a texture analyzer (TA.XT Express, SMS Ltd., Surrey, UK) equipped with an A/KIE probe, with each sample tested in triplicate, following the methodology adapted from Kim et al. [10]. The test parameters were set as follows: pre-test speed of 3 mm/s, test speed of 1 mm/s, and post-test speed of 5 mm/s. Tensile strength and elongation were calculated according to Equations (4) and (5).

(4) $$TS=\frac{F}{S}$$

(5) $$E=\frac{L_{1}-L_{0}}{L_{0}}$$

where *T_S_* is the tensile strength, MPa; *F* is the maximum force applied to the sample at the point of fracture, N; *S* is the cross-sectional area of the sample, mm^2^; *E* is the elongation, %; *L*_1_ is the total length of the sample after fracture, mm; and *L*_0_ is the length of the sample before fracture, mm.

#### 2.2.6. Determination of the Color of Yuba

The dried yuba was ground into powder using a small grinder and then placed into a self-sealing bag. The L*, a*, and b* values were measured at three randomly selected points using a colorimeter (Color i5, X Rite Corporation, Grand Rapids, MI, USA), following a methodology similar to that described by Qiu et al. [11].

#### 2.2.7. Determination of the Rehydration Ratio of Yuba

The yuba specimens with an initial mass (*m*_₁_) of 0.5 ± 0.02 g underwent controlled rehydration in distilled water maintained at 30 °C. During the 60 min rehydration process, samples were systematically monitored at 10-min intervals. At each timepoint, specimens were carefully removed from the aqueous medium, gently blotted with absorbent paper to remove excess surface moisture, and immediately weighed using an analytical balance (±0.1 mg precision). Following gravimetric analysis, the samples were promptly returned to fresh distilled water to maintain consistent hydration conditions. This measurement protocol was repeated for triplicate analytical replicates per experimental group to ensure methodological rigor. The rehydration ratio (*R*, %) was calculated following Equation (6).

(6) $$R=\frac{m_{2}}{m_{1}}$$

where *R* is the rehydration ratio, g/g; *m*_1_ is the initial mass of the yuba, g; and *m*_2_ is the mass of the yuba after rehydration, g.

#### 2.2.8. Determination of the Microstructure of Yuba

The microstructure of the yuba using different modification methods was observed using a scanning electron microscope (TM3030, JEOL Ltd., Tokyo, Japan), following the methodology adapted from Cai et al. [12]. For optimal imaging preparation, the yuba samples were carefully sectioned into 2 × 2 mm cross-sectional slices using a cryo-microtome maintained at −20 °C. The prepared specimens were then mounted on aluminum stubs using conductive carbon tape and subsequently sputter-coated with a 10 nm gold–palladium alloy layer (Q150T ES Plus, Quorum Technologies, Lewes, UK) to ensure surface conductivity. Imaging was performed under high vacuum conditions (5 × 10^−3^ Pa) using the secondary electron detection mode with an accelerating voltage of 15 kV and a working distance of 8 mm. Multiple representative regions from each sample were systematically examined across three biological replicates, with particular attention paid to the surface morphology and cross-sectional laminar structure at 200× magnification.

#### 2.2.9. Analysis of Thermal Stability of Yuba

The thermodynamic properties of the yuba were analyzed using differential scanning calorimetry (DSC, 823e, Mettler-Toledo Instruments Shanghai Co., Ltd., Shanghai, China) following a modified protocol adapted from Zhang et al. [6]. During sample preparation, the yuba samples were first dried at 60 °C to a constant weight to ensure that they were in an absolutely dry state. The dried samples were then ground into powder, and precisely 5.0 ± 0.1 mg of the powder was weighed and placed into hermetically sealed aluminum crucibles (40 μL capacity), with empty crucibles serving as reference controls. The samples were continuously heated from 20 to 275 °C at a constant rate of 5 °C/min under nitrogen purge (50 mL/min). Characteristic thermal parameters including glass transition temperature (*T*_g_), melting temperature (*T*_m_), and corresponding enthalpy change (Δ*H*) were calculated using STARe software v16.10 (METTLER-TOLEDO).

#### 2.2.10. Analysis of Protein Secondary Structure

This study adapted the method described by Aiello et al. [13], with slight modifications, for the determination of protein secondary structure. The yuba samples were homogenized in distilled water to obtain a protein solution (0.05 mg/mL). After vortex mixing (30 s) and static hydration (30 min at 4 °C), the suspension was centrifuged (CR22N, Hitachi, Tokyo, Japan) at 8000 rpm for 5 min at 4 °C. The supernatant was collected for subsequent analysis using a J-1500 circular dichroism spectrometer (Chirascan VX, Applied Photophysics Ltd., Leatherhead, UK) equipped with a temperature-controlled quartz cell (1 mm path length). Spectral measurements were performed in triplicate over the far-UV range (180–260 nm) with the following parameters: a bandwidth of 1 nm, a step size of 0.5 nm, and an acquisition time of 1 s per point. Three consecutive scans were averaged for each measurement to improve the signal-to-noise ratio. Prior to sample analysis, baseline correction was performed using matched distilled water as a blank control.

#### 2.2.11. Analysis of Protein Tertiary Structure

The yuba samples were homogenized and subsequently dissolved in distilled water to obtain a protein solution (0.05 mg/mL). After vortexing, the solution was subjected to centrifugation at 8000 rpm for 5 min to remove insoluble particulates. Fluorescence spectral characterization was performed using a Cary Eclipse fluorescence spectrophotometer (Agilent Technologies Inc., Santa Clara, CA, USA) equipped with a temperature-controlled cell holder. The excitation wavelength was set at 280 nm with a 10 nm slit width, while emission spectra were acquired from 300 to 450 nm using matched slit configurations. All measurements were conducted under controlled ambient conditions (25 ± 0.5 °C) with a scanning rate of 600 nm/min. To ensure data reproducibility, three consecutive scans were performed for each independent sample preparation, with baseline correction applied using solvent blanks.

#### 2.2.12. Sensory Evaluation of Yuba

This study was supervised by the School of Food and Bioengineering at Henan University of Science and Technology and conducted in strict accordance with the *Declaration of Helsinki*. The sensory evaluation component of the experiment involved only low-risk sensory testing of food samples, with no invasive procedures or experiments posing health risks to participants. All participants voluntarily participated in the study after signing a written informed consent form, fully understanding the purpose, content, procedures, and potential risks of the experiment. The design and implementation of the experiment were supervised and reviewed by the school. As it was classified as a low-risk study, no specific ethical approval number was issued. The study adhered strictly to principles of participant privacy protection, with all data anonymized and used solely for research purposes. Both the experimental process and the rights of participants were rigorously safeguarded, fully complying with relevant ethical standards. A panel of 20 trained sensory evaluators (10 male and 10 female, aged 18–45 years) was convened following standardized selection criteria to conduct quantitative evaluation of yuba’s sensory attributes using standardized methodology. Prior to sensory analysis, the specimens underwent standardized preparation as follows: reconstituted through boiling in distilled water (100 °C) for 5 min, followed by rapid cooling to 25 °C. The panel established comprehensive evaluation criteria (Table 2), incorporating both intensity scoring and descriptive analysis of textural parameters, with particular emphasis on chew resistance measurement under controlled mastication conditions (3 s interval between bites).

### 2.3. Statistical Analysis

All experimental procedures were conducted in triplicate to ensure methodological reproducibility. Statistical analyses were performed using SPSS 26 (IBM Corp., Armonk, NY, USA), with additional data processing carried out in Microsoft Excel 2021 (version 16.0) and graphical representations created using OriginPro 2023 (v9.9.0.225, OriginLab Corporation, Northampton, MA, USA). For parametric data comparisons, a one-way analysis of variance (ANOVA) followed by Tukey’s post hoc test was employed for intergroup comparisons, with statistical significance defined as a two-tailed *p*-value < 0.05 determined a priori.

## 3. Results and Discussion

### 3.1. Effects of Protein Modification Methods on the Drying Characteristics of Yuba

**Figure 1:**
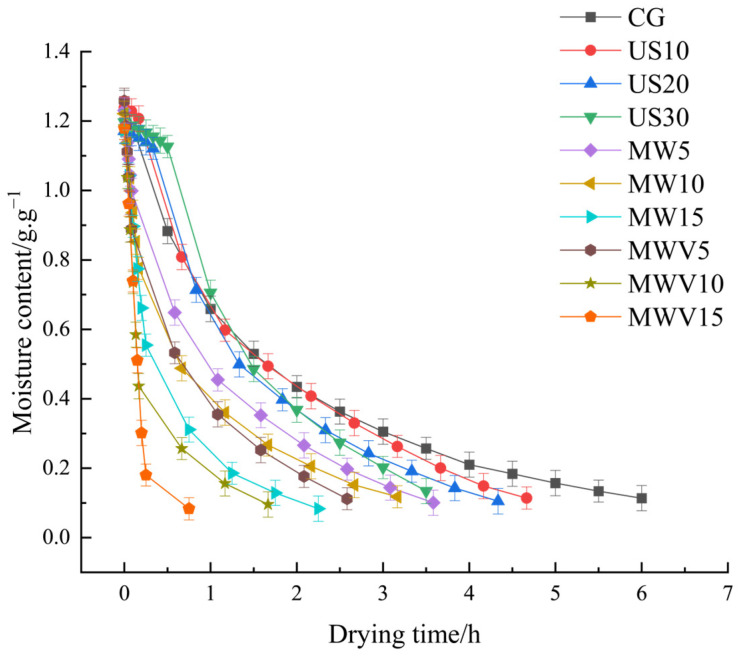
Drying curves of yuba treated with different protein modification methods.

**Figure 2:**
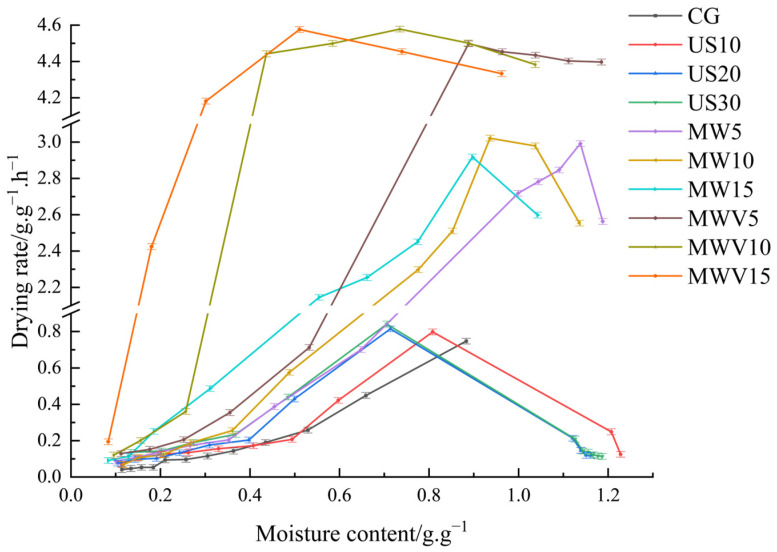
Drying rate curves of yuba under different protein modification methods.

The drying curve revealed significant acceleration of moisture migration through modified protein matrices (Figure 1). After protein modification treatments, the drying time required to reach the target moisture content decreased. With the increase in ultrasound treatment time, the drying time of the yuba continued to decrease. The drying process in the US30 group was 2.50 h shorter than that of the CG group. This reduction can be attributed to the cavitation effect induced by ultrasound pretreatment, which created micro-pores within the yuba, thereby enhancing the diffusion channels for moisture and accelerating its migration and evaporation. Although this effect was most pronounced during the initial drying phase, it fundamentally altered the internal structure of the yuba, enabling more efficient moisture migration. The enhanced moisture diffusion pathways immediately influenced the drying kinetics, leading to accelerated evaporation at the early stage and exerting a sustained impact on the overall drying performance [7]. During the microwave and microwave–vacuum treatment processes, the thermal effect of microwaves rapidly heats the interior of the material, generating steam pressure and creating micropores or cracks. This accelerates the evaporation and migration of moisture, leading to a significant increase in the drying rate [8]. Particularly in microwave–vacuum treatment (MWV), the vacuum environment lowers the boiling point of moisture and reduces air resistance, allowing moisture to evaporate more quickly [9]. As a result, the drying rate significantly improved, and the drying time for the yuba in the MWV15 group to reach the target moisture content was only 0.75 h. As shown in Figure 2, with the same dry matter moisture content, the drying rate of the yuba after three protein modification treatments improved, indicating that the three protein modification methods had an effect on the microscopic structure of the yuba. Ultrasonic treatment disrupts the cell walls or fibrous structures of a material [2], creating more channels for moisture migration, which causes a sudden acceleration in the drying rate. On the other hand, microwave treatment rapidly heats the interior of the material, forming micropores or cracks, which further accelerates moisture evaporation. Microwave–vacuum treatment further enhances this process because the vacuum environment lowers the boiling point of moisture, thereby speeding up the evaporation and migration of moisture. These modification methods caused protein denaturation, resulting in changes in the microstructure of the yuba, which improved its moisture migration ability and, consequently, increased the drying rate.

### 3.2. Effects of Protein Modification Methods on the Mechanical Properties and Color of Yuba

As shown in Table 3, the three protein modification methods—ultrasound, microwave, and microwave–vacuum—improved the tensile strength of the yuba, with microwave modification having the strongest effect and ultrasound modification having the weakest. The tensile strength of the yuba in the MW15 group was the highest, at 5.22 MPa, but at this point, the elongation was the lowest, at only 6.63%, and the color was dark, with an L* value of 58.66. The yuba in the MWV10 group exhibited good tensile strength and elongation, with values of 4.21 MPa and 10.41%, respectively, and a better color, with an L* value of 64.98. The mechanical vibration of ultrasound can cause protein molecules in the yuba to unfold, exposing internal active sites, which helps form new hydrogen bonds and van der Waals interactions, increasing crosslinking between molecules and thus improving the tensile strength of the yuba [14]. Since wet yuba contains a significant amount of water, cavitation effects occur when ultrasound propagates through the liquid. The microbubbles produced during cavitation cause localized increases in temperature and pressure upon collapsing, which also leads to changes in protein structure [15]. The thermal effect of microwaves provides heat energy to the protein molecules in the yuba, accelerating molecular motion and promoting the formation of intermolecular crosslinks, thus enhancing the stability of the protein network and increasing tensile strength. However, prolonged microwave treatment causes rapid moisture loss in yuba, resulting in an excessively high crosslinking density, making the protein network tighter and reducing the elongation of the yuba [16]. During microwave treatment, the Maillard reaction occurs between sugars and proteins in the yuba, resulting in a deeper color, which intensifies with increased modification time. Furthermore, prolonged microwave heating leads to the decomposition of some proteins and sugars, producing substances with a darker color, which also causes a decrease in the L* value of the yuba. Microwave–vacuum treatment allows microwaves to penetrate the yuba sample more evenly, ensuring uniform heating. Additionally, under vacuum conditions, moisture evaporates more easily. This rapid and uniform moisture evaporation prevents local overheating and avoids collapse of the yuba’s tissue structure, helping it maintain a good network structure with better tensile strength and elongation. The reduced oxygen in the vacuum environment significantly slows down the oxidation rate, which helps preserve the yuba’s color [17].

### 3.3. Effects of Protein Modification Methods on the Rehydration Properties of Yuba

**Figure 3:**
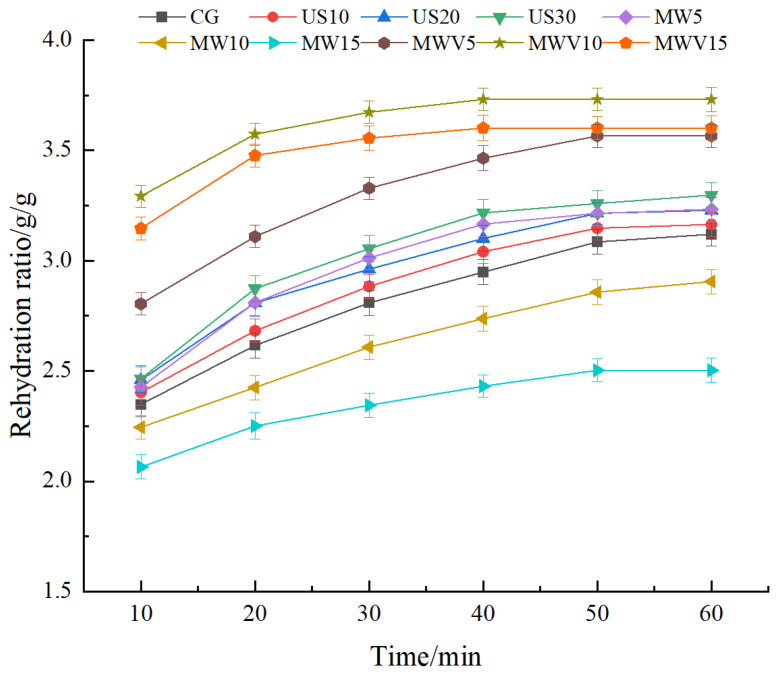
Effect of protein modification methods on the rehydration properties of yuba.

As shown in Figure 3, the rehydration performance of yuba treated with ultrasound and microwave–vacuum improved compared to untreated yuba, with microwave–vacuum treatment having a superior effect on rehydration performance compared to ultrasound treatment. The rehydration performance of yuba in the MWV10 group was the best, while MW5 treatment improved rehydration performance, but the rehydration performance of yuba treated with MW10 and MW15 decreased significantly compared to the untreated yuba. The microbubbles formed during ultrasound treatment create pore structures when they collapse. These pores can serve as channels for water molecules during the rehydration process, enhancing both the rehydration rate and the amount of water absorbed [18]. During microwave–vacuum treatment, the steam pressure generated inside the yuba during moisture migration causes its volume to expand, preserving its loose and porous structure and thus improving rehydration performance [19]. Short-term microwave treatment alters the protein network structure, exposing more hydrophilic groups, which increases the hydrophilicity of the protein and improves its rehydration ability. However, prolonged microwave treatment leads to an overly tight protein network structure, and the rapid evaporation of moisture causes the protein structure to collapse, reducing its rehydration performance [20].

### 3.4. Effects of Protein Modification Methods on the Microstructure of Yuba

**Figure 4:**
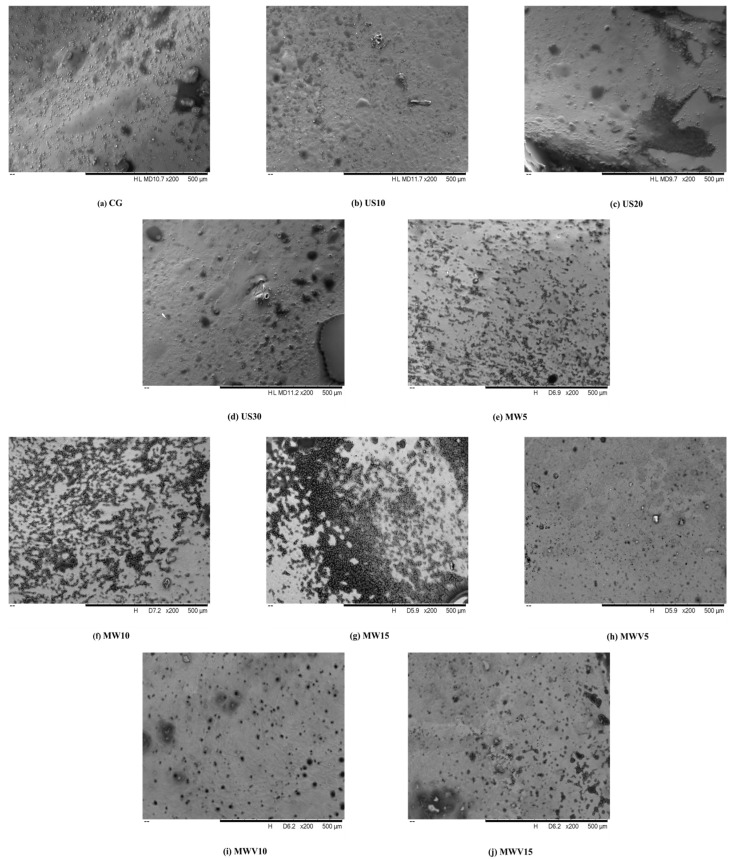
Effects of protein modification methods on the microstructure of yuba.

The surface microstructure of yuba obtained using different protein modification methods is shown in Figure 4. From the figure, it can be seen that the surface of yuba treated with ultrasound has obvious pores, and as the ultrasound treatment time increases, the number of pores on the surface also increases. This is because during the ultrasound treatment of wet yuba, cavitation occurs, generating small bubbles inside the yuba tissue. With continuous ultrasound treatment, these small bubbles burst, creating fine pores in the yuba tissue [21]. The surface of yuba treated with microwave shows darkening, and as the microwave treatment time increases, the darkened areas become more pronounced. This is because the high moisture content in wet yuba causes water molecules to vibrate rapidly during microwave heating. The heat generated causes the internal temperature of the yuba to rise quickly, and the uneven distribution of moisture leads to localized overheating. Under such conditions, the proteins, sugars, and other components in the yuba undergo Maillard reactions, resulting in the formation of dark-colored substances on the surface [22]. Observing the surface microstructure of yuba treated with microwave–vacuum, it can be seen that there are evenly distributed pores on the surface, with no obvious darkening. This is because, in a vacuum environment, the uniformity of microwave heating is improved, helping to avoid local overheating and reducing the occurrence of Maillard reactions. In addition, due to the reduced oxygen content in the vacuum environment, the Maillard reaction is inhibited, which helps preserve the original color of the yuba [23].

### 3.5. Effects of Protein Modification Methods on the Thermal Stability of Yuba

Table 4 shows the glass transition temperature (*T*_g_), melting temperature (*T*_m_), and enthalpy change (∆*H*) of yuba treated with different protein modification methods. The results indicate that the thermal stability of the yuba varies after protein modification, which may be due to differences in the protein structure and conformation. As shown in the table, the *T*_g_ and *T*_m_ of yuba increase with the treatment time, while ∆*H* decreases with the increase in treatment time. Ultrasound treatment has the least impact on the thermal stability of the yuba, while microwave treatment has the greatest effect, and microwave–vacuum treatment has a slightly smaller effect than microwave treatment. Yuba treated with ultrasound, microwave, and microwave–vacuum modification methods all exhibit higher *T*_g_ values, indicating that more energy is required to break the molecular chains as the yuba transitions from an elastic state to a glassy state. Higher *T*_g_ values indicate slower molecular movement, where protein aggregation requires large-scale diffusion. Yuba with higher *T*_g_ values tend to form an amorphous matrix (glassy state) during modification, which fixes the protein molecules, making the protein structure denser and inhibiting aggregation. Furthermore, studies have shown that protein stability can be maintained when materials stored at temperatures below *T*_g_. A higher *T*_g_ value indicates higher thermal tolerance and stability at higher temperatures [24]. Therefore, modified yuba can be stored at higher temperatures while maintaining its stability. The increase in *T*_m_ is due to the enhancement of crosslinking between protein molecules, which reduces the molecular mobility of the yuba. The cleavage of disulfide bonds and the formation of new inter-chain disulfide bonds stabilize the protein structure, restricting the movement of protein chains. Breaking the protein structure under these conditions requires more energy, and the stronger intermolecular forces increase the stability of the yuba [25]. ∆*H* is related to the number of ordered secondary structures in the protein. Generally, higher ∆*H* values indicate more hydrophilic and hydrophobic interactions, leading to lower thermal stability [26]. The effect of microwave–vacuum treatment on ∆*H* is less than that of microwave treatment because microwave–vacuum treatment results in a loose and porous structure in yuba, reducing its thermal resistance and thus lowering its thermal stability.

### 3.6. Effects of Protein Modification Methods on the Secondary Structure of Yuba Protein

**Figure 5:**
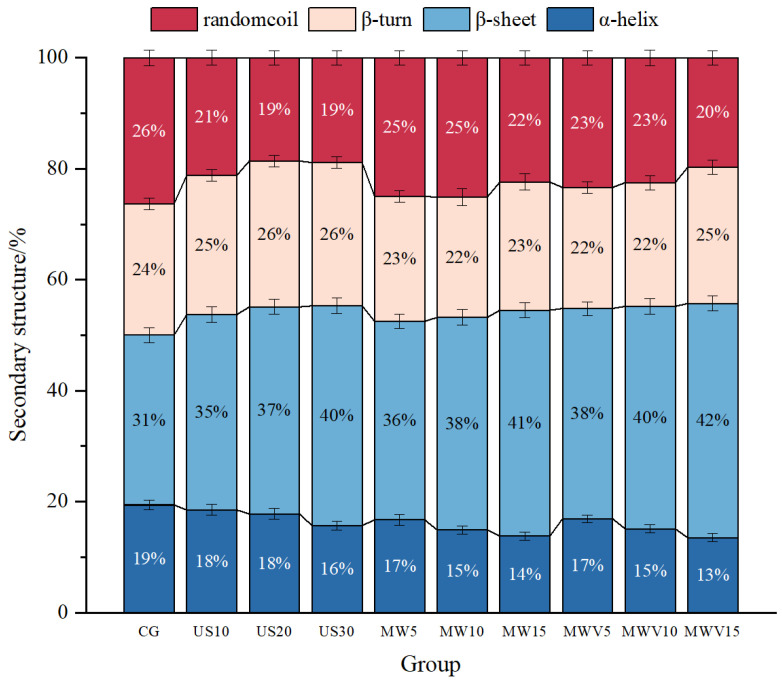
Effect of protein modification methods on the secondary structure content of yuba protein.

As shown in Figure 5, after treatment with the three protein modification methods, the *α*-helix content in the protein secondary structure of yuba shows a continuous decrease, while the *β*-sheet content exhibits a continuous increase. However, microwave and microwave–vacuum treatments have a more significant effect on the *α*-helix content. After 30 min of ultrasound treatment, the *α*-helix content decreases significantly. Yuba treated with microwave–vacuum has the highest *β*-sheet content in its protein secondary structure. Ultrasound treatment affects the protein molecular structure through mechanical vibration and cavitation effects, but this effect is relatively weak. As a result, a significant decrease in *α*-helix content only occurs after 30 min of treatment. The localized high temperature and pressure differences caused by ultrasound may induce changes in the hydrogen bonds and hydrophobic interactions in protein molecules, leading to the conversion of *α*-helices and random coils to *β*-sheets and *β*-turns [27]. Microwave and microwave–vacuum treatments, on the other hand, utilize the thermal effect generated by microwave radiation to alter the protein structure [28]. Since microwaves can penetrate yuba and generate heat throughout the sample, the rapid vibration of protein molecules increases the intermolecular distance, thereby disrupting hydrogen bonds and hydrophobic interactions. This causes the *α*-helix structure to unravel and rearrange into *β*-sheet structures [29]. Compared to microwave treatment at atmospheric pressure, microwave–vacuum treatment in a vacuum environment lowers the boiling point of water, which promotes more heat-induced conformational changes in proteins. As a result, it shows a higher increase in *β*-sheet content [23]. This structural change helps improve the tensile strength and water retention capacity of yuba, thereby enhancing its texture. These findings are consistent with the results in Section 3.2 and Section 3.3.

### 3.7. Effects of Protein Modification Methods on the Tertiary Structure of Yuba Protein

**Figure 6:**
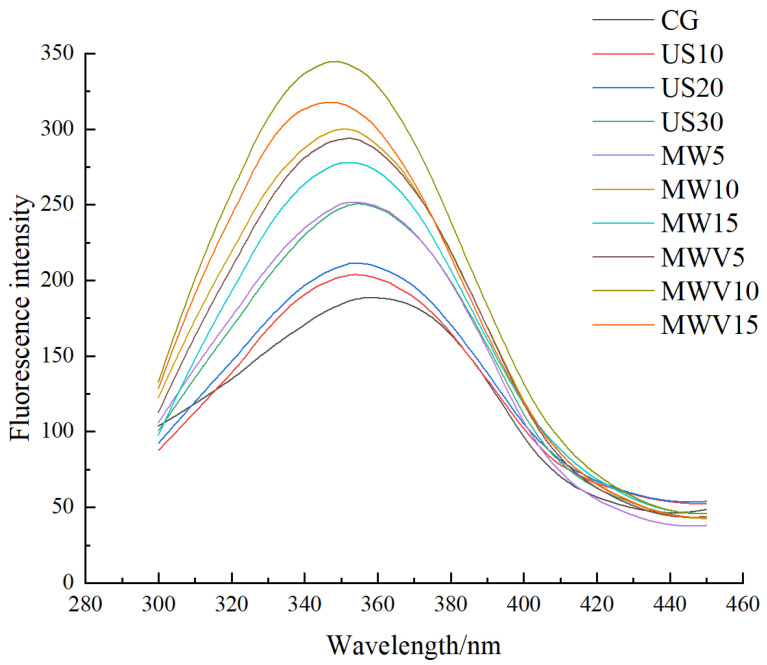
Effect of protein modification methods on the fluorescence spectrum of yuba.

The fluorescence spectra of yuba subjected to different protein modification methods are shown in Figure 6. Tryptophan in the protein can emit fluorescence under ultraviolet light excitation, so the maximum emission wavelength and fluorescence intensity in the endogenous fluorescence spectra reflect changes in the tryptophan residues within the protein’s tertiary structure [30]. As seen in the figure, after treatment with the three protein modification methods, the fluorescence intensity of the yuba increased, with the microwave–vacuum treatment showing the strongest enhancement, and the ultrasound treatment showing the weakest effect. As the ultrasound treatment time increases, the fluorescence intensity continuously increases. However, for microwave and microwave–vacuum treatments, the fluorescence intensity first increases and then decreases as the treatment time increases, with the MWV10 group reaching the highest fluorescence intensity. This phenomenon occurs because the three modification methods affect the protein structure in different ways. Ultrasound treatment typically induces cavitation effects and shear forces, leading to the unfolding of the protein structure and exposing the tryptophan residues, thus increasing fluorescence intensity [31]. The thermal effect produced by microwave treatment provides energy for the movement of protein molecules, causing severe disruption of the protein structure and exposing more tryptophan residues, which results in a significant increase in fluorescence intensity. However, prolonged microwave treatment leads to protein aggregation, which decreases fluorescence intensity.

### 3.8. Effects of Protein Modification Methods on Sensory Evaluation of Yuba

**Figure 7:**
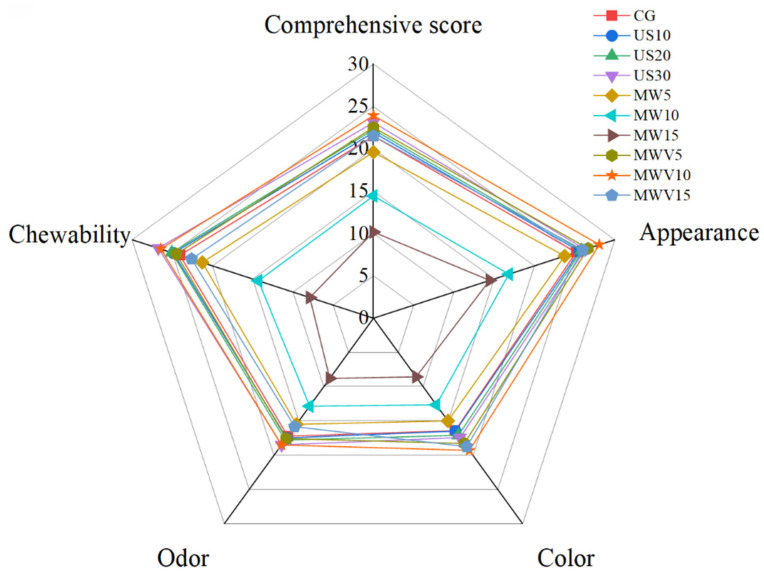
Effect of protein modification methods on the sensory evaluation of yuba.

The impact of protein modification methods on the sensory evaluation of yuba is shown in Figure 7. As seen in the figure, the MWV10 group of yuba received the highest overall score, with good results in all sensory evaluation indicators, while the MW15 group received the lowest overall score. The sensory scores of yuba treated with ultrasound and microwave–vacuum modification were improved, while the sensory scores of yuba treated with microwave processing decreased to varying degrees. This is because yuba treated with ultrasound and microwave–vacuum modification maintain a good protein network structure, while prolonged microwave treatment leads to excessive cross-linking of the protein, resulting in deterioration of the texture and promoting the Maillard reaction, which causes the color to darken. Therefore, the MWV10 modification method not only helps the yuba retain a good appearance and aroma but also improves its texture.

## 4. Conclusions

This study systematically investigated the effects of physical field protein modification methods on the mechanical properties, color, rehydration performance, thermal stability, and sensory quality of yuba. The results showed that the drying time for the US30 group was shortened by 2.5 h, while the drying time for the MWV15 group was only 0.75 h. Among them, the MWV10 group of yuba exhibited the best comprehensive performance, with a tensile strength of 4.21 MPa, an elongation at break of 10.41%, favorable color (L* value of 64.98), and the highest sensory score. Additionally, this group demonstrated excellent rehydration performance and thermal stability, with a rehydration ratio of 3.73 g/g, a faster rehydration rate, a glass transition temperature (*T*g) of 85.74 °C, and an enthalpy (∆*H*) of 36.31 J/g. These superior properties can be attributed to the unique advantages of microwave treatment under vacuum conditions, where microwaves uniformly penetrate the yuba, ensuring even heat distribution throughout the material, while the reduced boiling point in the vacuum state accelerates moisture evaporation, thereby preventing local overheating and structural collapse and allowing the yuba to maintain an excellent network structure, resulting in high-quality yuba. These findings were further supported by studies on the microstructure and secondary and tertiary protein structures of the yuba. This research provides a theoretical basis and technical support for the application of physical field modification technology in yuba processing, with significant implications for improving yuba quality and promoting industrial development.

## Figures and Tables

**Table 1:** Protein modification strategies.

Group	Modification Method
CG	Hot air drying at 60 °C until the moisture content is about 10%
US10	After 100 W ultrasonic treatment for 10 min, hot air drying at 60 °C on a wet basis with moisture content of about 10%
US20	After 100 W ultrasonic treatment for 20 min, hot air drying at 60 °C on a wet basis moisture content of about 10%
US30	After 100 W ultrasonic treatment for 30 min, hot air drying at 60 °C on a wet basis with moisture content of about 10%
MW5	Microwave power 3 W/g for 5 min, then hot air drying at 60 °C on a wet basis with moisture content of about 10%
MW10	Microwave power 3 W/g for 10 min, then hot air drying at 60 °C on a wet basis with moisture content of about 10%
MW15	Microwave power 3 W/g for 15 min, then hot air drying at 60 °C on a wet basis with moisture content of about 10%
MWV5	Microwave power 3 W/g and vacuum degree −0.1 MPa treatment for 5 min and then hot air drying at 60 °C on a wet basis with moisture content of about 10%
MWV10	Microwave power 3 W/g and vacuum degree −0.1 MPa treatment for 10 min and then hot air drying at 60 °C on a wet basis with moisture content of about 10%
MWV15	Microwave power 3 W/g and vacuum degree −0.1 MPa treatment for 15 min and then hot air drying at 60 °C on a wet basis with moisture content of about 10%

**Table 2:** Sensory evaluation standards.

Types	Points	Evaluation Standards
Appearance	30	Even branches, flat surface, and complete structure, 21–30 points; partially broken branches, rough surface, and damaged structure, 11–20 points; rough and incomplete surface and damaged structure, 0–10 points
Color	20	The color is bright and light yellow with natural oily luster, 16–20 points; the color is darker and basically dull, 11–15 points; the color is dark yellow or yellowish brown, and the color is dull, 0–10 points
Odor	20	Fresh and rich soybean aroma, no odor, 16–20 points; light soybean aroma, slight odor, 11–15 points; no soybean aroma, strong odor, 0–10 points
Chewability	30	Good chewability and good taste, 21–30 points; poor chewability and chewing is more laborious, 11–20 points; poor chewability and chewing is laborious, 0–10 points

**Table 3:** Effects of protein modification methods on the mechanical properties and color of yuba.

Group	Tensile Strength/MPa	Elongation/%	L*	a*	b*
CG	3.54 ± 0.19	12.61 ± 0.61	66.74 ± 0.43	−1.64 ± 0.08	7.27 ± 1.03
US10	3.72 ± 0.26	13.29 ± 0.41	67.08 ± 0.45	−2.15 ± 0.26	6.70 ± 0.80
US20	3.83 ± 0.26	13.17 ± 0.36	68.01 ± 0.55	−2.16 ± 0.10	7.45 ± 0.63
US30	3.91 ± 0.29	12.82 ± 0.67	69.05 ± 1.32	−2.70 ± 0.21	6.01 ± 0.78
MW5	4.29 ± 0.28	10.90 ± 0.76	60.65 ± 0.58	2.31 ± 0.20	7.35 ± 0.14
MW10	4.84 ± 0.26	7.92 ± 0.40	60.44 ± 0.57	2.24 ± 0.33	8.24 ± 0.41
MW15	5.22 ± 0.23	6.63 ± 0.55	58.66 ± 0.41	1.94 ± 0.30	10.51 ± 0.79
MWV5	3.92 ± 0.39	11.48 ± 0.35	66.59 ± 0.95	−1.91 ± 0.27	6.00 ± 0.17
MWV10	4.21 ± 0.25	10.41 ± 0.59	64.98 ± 0.62	−1.92 ± 0.42	5.75 ± 0.60
MWV15	4.43 ± 0.30	8.47 ± 0.57	63.87 ± 0.68	−2.60 ± 0.35	7.55 ± 1.33

**Table 4:** Effect of protein modification methods on the thermodynamic parameters of yuba.

Group	*T*_g_ (°C)	*T*_m_ (°C)	∆*H* (J/g)
CG	81.52 ± 0.66	172.33 ± 0.91	45.50 ± 0.66
US10	82.32 ± 0.78	172.77 ± 0.63	42.20 ± 0.73
US20	83.67 ± 0.71	173.39 ± 0.58	40.26 ± 0.73
US30	85.13 ± 1.12	174.63 ± 0.65	37.72 ± 0.70
MW5	84.34 ± 0.55	174.22 ± 1.27	38.83 ± 0.83
MW10	87.27 ± 0.81	178.03 ± 0.79	33.77 ± 0.69
MW15	89.13 ± 0.64	181.57 ± 1.10	30.79 ± 0.66
MWV5	84.46 ± 0.67	173.50 ± 0.86	40.68 ± 0.78
MWV10	85.74 ± 0.67	177.27 ± 0.86	36.31 ± 0.59
MWV15	87.67 ± 0.74	180.37 ± 0.86	33.77 ± 0.73

## Data Availability

The original contributions presented in the study are included in the article, further inquiries can be directed to the corresponding author.
